# In vivo assembly of epitope-coated biopolymer particles that induce anti-tumor responses

**DOI:** 10.1038/s41541-023-00787-8

**Published:** 2024-01-23

**Authors:** Devi Jenika, Saranya Pounraj, David Wibowo, Leonhard M. Flaxl, Bernd H. A. Rehm, Justine D. Mintern

**Affiliations:** 1 Department of Biochemistry and Pharmacology, The University of Melbourne, Bio21 Molecular Science and Biotechnology Institute, Parkville, VIC 3010 Australia; 2 Centre for Cell Factories and Biopolymers, Griffith Institute for Drug Discovery, Griffith University, Nathan, QLD 4111 Australia; 3 Menzies Health Institute Queensland, Griffith University, Southport, QLD 4215 Australia

**Keywords:** Vaccines, Adaptive immunity, Tumour immunology

## Abstract

There is an unmet need for antigen delivery systems that elicit efficient T cell priming to prevent infectious diseases or for treatment of cancers. Here, we explored the immunogenic potential of biologically assembled biopolymer particles (BPs) that have been bioengineered to display the antigenic MHC I and MHC II epitopes of model antigen ovalbumin (OVA). Purified dendritic cells (DCs) captured BP-OVA and presented the associated antigenic epitopes to CD4^+^ T cells and CD8^+^ T cells. Vaccination with BP-OVA in the absence of adjuvant elicited antigen presentation to OVA-specific CD8^+^ and CD4^+^ T cells and cross-primed effective cytotoxic T lymphocyte (CTL) killers. BP-OVA induction of CTL killing did not require CD4^+^ T cell help, with active CTLs generated in BP-OVA vaccinated *I-A*^*b*−/−^ and *CD40*^−/−^ mice. In contrast, IL-15 and type I IFN were required, with abrogated CTL activity in vaccinated *IL-15*^−/−^ and *IFNAR1*^−/−^ mice. cDC1 and/or CD103^+^ DCs were not essential for BP-OVA specific CTL with immunization eliciting responses in *Batf3*^−/−^ mice. Poly I:C, but not LPS or CpG, co-administered as an adjuvant with BP-OVA boosted CTL responses. Finally, vaccination with BP-OVA protected against B16-OVA melanoma and Eμ-myc-GFP-OVA lymphoma inoculation. In summary, we have demonstrated that epitope-displaying BPs represent an antigen delivery platform exhibiting a unique mechanism to effectively engage T cell immune responses.

## Introduction

Advances in the design of safe and effective vaccine delivery systems is of critical importance. Bacterially assembled biopolymer particles (BPs) bioengineered to display pathogen-specific antigens were shown in preclinical studies to perform as safe and efficient vaccines^1–4^. BPs are composed of the biopolyester, polyhydroxybutyrate (PHB) that has Federal Drug Administration, USA-approved uses including for sutures, implants and tissue engineering^4,5^. PHB inclusions are naturally produced by bacteria and archaea as an energy storage material^1^ and are polydisperse with a size range of 200 to 500 nm in diameter^2^. BPs can be isolated from bacterial cells and are structurally stable, biocompatible and biodegradable. An attractive property of BPs is their capacity to be bioengineered to display functional proteins and epitopes of interest^1,6^. This is achieved by exploiting the PHB synthesizing enzyme, which mediates BP formation, to anchor translationally fused antigens or epitopes to the surface^1^. Thus, antigens are biologically conjugated to BPs during their assembly by the bacterial production host. BP prototypes displaying antigens or epitopes of interest can be developed within weeks and subjected to a scalable industrial production process^4^. These features lend BPs many attributes that position them as ideal antigen carrier systems.

Successful immunogenicity is evident when BPs engineered to display pathogen-associated antigens are administered subcutaneously or intramuscularly. BPs displaying individual or multiple *Mycobacterium* *tuberculosis*-specific antigens provoked inflammatory cytokine production and antibody responses that correlated with protective immunity^7–9^. When displayed by BPs, *Streptococcus pneumoniae* antigen PsaA^10^ and *N. meningitidis* antigen NadA^11^ induce strong antibody responses. Display of epitopes derived from SARS-CoV-2 spike protein or *Plasmodium falciparum* induced T cell immunity in respective animal models^12,13^. While these examples highlight the potential of BPs as vaccines, the parameters of how BPs elicit T cell immunity are yet to be investigated and their utility in tumour immunity unexplored. Here, we have undertaken an in-depth examination of the immunogenic functions of BPs. We show BPs are capable of mediating MHC I and MHC II antigen presentation, T cell priming and tumour regression in mouse models of cancer immunotherapy.

## Results

### Design and production of OVA-peptide coated BPs

**Fig. 1:**
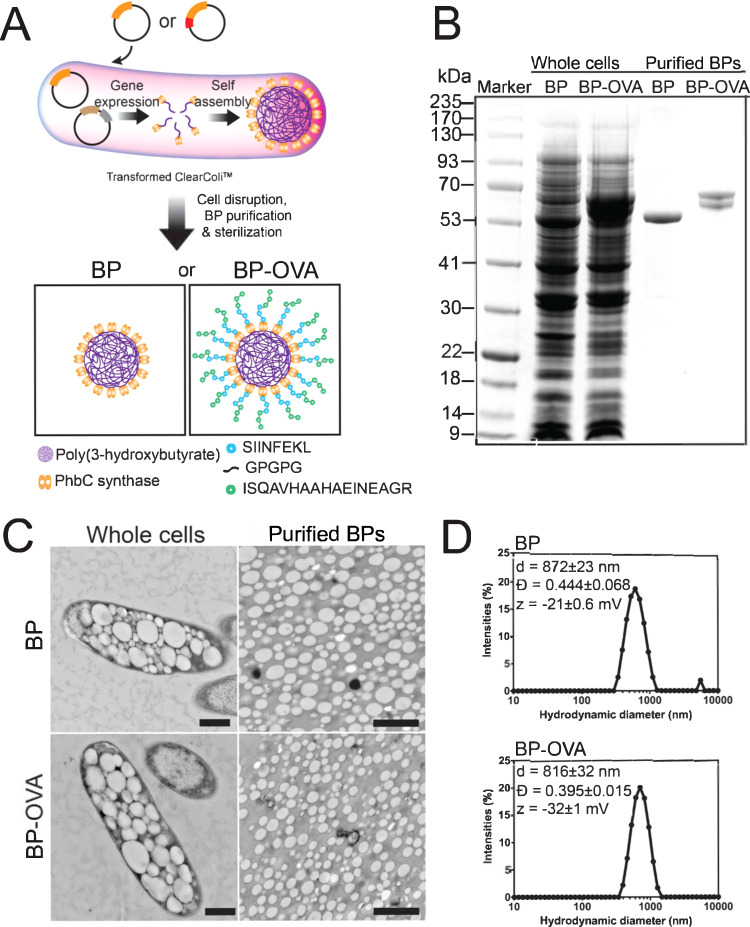
Design, synthesis and characterization of bioengineered poly(3-hydroxybutyrate) beads displaying ovalbumin peptides. **A** Schematic illustration of bioengineering *ClearColi*^TM^, an endotoxin-free *Escherichia coli* BL21(DE3) strain. The individual cells harbor two plasmids encoding for PhaA (brown) and PhaB (gray) on one plasmid, as well as encoding for PhbC synthase (orange) on the other plasmid or its translationally fusion to both SIINFEKL and ISQAVHAAHAEINEAGR (BP-OVA, red). Synergistic functionalities of the expressed genes enable in vivo production of a library of BPs. **B** Protein profiles of whole cells containing BPs and of purified BPs as determined by sodium dodecyl sulfate gel electrophoresis (SDS-PAGE). **C** Transmission electron microscopy (TEM) images of harvested cells (left panel; scale bar, 500 nm) and of purified BPs (right panel; scale bar, 2 μm). **D** Size distribution of BPs dispersed in 10 mM Tris buffer pH 7.5 obtained based on dynamic light scattering, along with their Z-average diameter (*d*), dispersity (Ð) and zeta potential (ζ).

BPs displaying multiple repeats of peptides derived from the model antigen ovalbumin (OVA) were synthesized. This was performed by engineering self-assembly of BPs in *ClearColi*^TM^, a mutant strain of *Escherichia coli* BL21(DE3) incapable of producing endotoxic lipopolysaccharides (Fig. 1A). Self-assembly of BPs involved genetic engineering of *ClearColi*^TM^ to enable biosynthesis of PHB precursors and the formation of OVA-peptide coated BP^14^ (Supplementary Tables 1 and 2). Using this method, synthesis and peptide functionalization of BPs was integrated in a single step in microbial cell factories with the capacity for manufacture to be easily scaled up. To display OVA on BPs, three repeats of OVA_257-264_ (MHC I specific epitope) and OVA_323-332_ (MHC II specific epitope) peptides were fused to the N terminus of PHB synthase (PhbC), the PHB anchoring protein. T cell epitopes were repeated to promote effective antigen presentation and T cell responses. The N-terminus fusion point of PhbC was selected due to its highly variable surface-exposed region which is not critical for PhbC activity^15^. This fusion point is also expected to prevent unfavorable interactions between the OVA peptide domains and PHB or PhbC, while allowing unconstrained access of the peptides for antigen processing. Protein profiles of the BPs after processing indicated BP purity when compared to whole cells harbouring BPs and showed the presence of the fusion proteins of PHB anchor and OVA-peptides corresponding with the expected molecular weight, larger than that of only the PHB anchor or the empty control BPs (Fig. 1B). Sequences of the fusion proteins were confirmed through peptide fingerprinting analyses combined with mass spectrometry (Supplementary Table 3), suggesting successful production of BPs densely displaying the respective OVA peptides. It should be noted that the doublet in the OVA peptide-coated BP sample evident in the gel electrophoresis (Fig. 1B) was confirmed to comprise the same amino acid sequence (Supplementary Table 3). Further compositional analyses of the BPs after acid-digestion of PHB into crotonic acid confirmed the presence of PHB (Supplementary Table 3). Transmission electron microscopy (TEM) images showed inclusions of BPs in whole cells, resulting in different aspect ratios of width and length (Fig. 1C, left panel), as well as the purified BPs (Fig. 1C, right panel). Although BPs showed a size of 100-200 nm in TEM, their hydrodynamic diameters in suspensions were approximately 800–900 nm when determined by dynamic light scattering (Fig. 1D). This suggested aggregation in suspension. The BPs showed negative zeta potentials. Overall, this process generated BPs densely coated with OVA peptides referred to throughout the manuscript as BP-OVA.

### BPs are weakly immunogenic

**Fig. 2:**
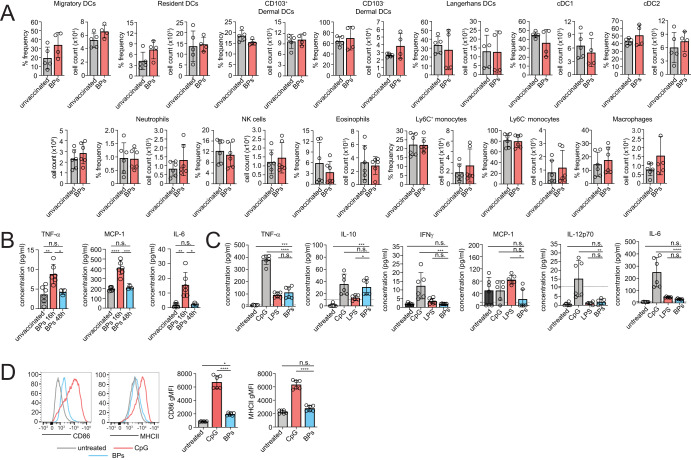
BPs are weakly immunogenic. **A** C57BL/6 mice were subcutaneously injected with 5 mg BPs or were unvaccinated. Inguinal lymph nodes were harvested after 16 h to determine the frequency and total number of immune cells. **B** C57BL/6 mice were subcuteneously injected with 5 mg BP-OVA and sera analysed by BD cytometric bead array after 16 or 48 h. **C** Splenic DCs were purified from C57BL/6 mice and incubated for 24 h in the presence of 0.5 μM of CpG type B 1688, 0.1 μg/ml O127:B8 *E. coli* LPS or 100 μg BP at 37 °C. Supernatant and serum were collected and cytokines analysed by BD Cytometric Bead Array (CBA) Mouse Inflammation Kit for flow cytometry analysis using FCAP^TM^ Array Software. **D** MuTu DCs were incubated with or without 0.5 μM of CpG type B 1688 or 100 μg BP for 24 h at 37 °C prior to analysing surface levels of CD86 and MHC II by flow cytometry. Data is pooled from 1 - 2 independent experiments performed in triplicate. Bars represent mean ± SD and analysed using one-way ANOVA with Tukey’s multiple comparison test. *p < 0.05, **p < 0.01, ***p < 0.001, ****p < 0.0001, n.s. = not significant.

First, the ability of BPs to elicit inflammatory responses following administration in vivo was determined. Mice were injected subcutaneously with 5 mg of BPs that did not display any fusion proteins. Examination of immune cells in the subcutaneous inguinal lymph node (ILN) 16 h after immunisation showed the overall proportion and numbers of innate and adaptive immune cells (for gating strategy refer to Supplementary Fig. 1), including DCs, neutrophils, NK cells, eosinophils, monocytes and macrophages, were unaltered compared to unvaccinated mice (Fig. 2A). While BP immunization did not perturb the cellular composition of the lymph node, immunisation elicited a temporary increase in serum TNF-α, MCP-1, and IL-6 at 16 hours post injection, with responses returning to basal levels after 48 hours (Fig. 2B). IL-10, IL-12p70 or IFNγ was not detected (data not shown). BPs directly incubated with primary DCs isolated from murine spleens induced small amounts of TNF-α and IL-10, but not IFNγ, MCP-1, IL-12p70 or IL-6. The pattern of cytokines elicited by BPs differed to that following stimulation with either synthetic unmethylated CpG oligonucleotides (CpG) or lipopolysaccharide (LPS) (Fig. 2C). Finally, BPs caused a small, but detectable increase in MuTuDC surface expression of costimulatory molecule CD86, while MHC II was unchanged when compared with MuTuDCs stimulated with CpG (Fig. 2D).

### DCs present antigen displayed by BPs to CD8^+^ and CD4^+^ T cells in vitro

**Fig. 3:**
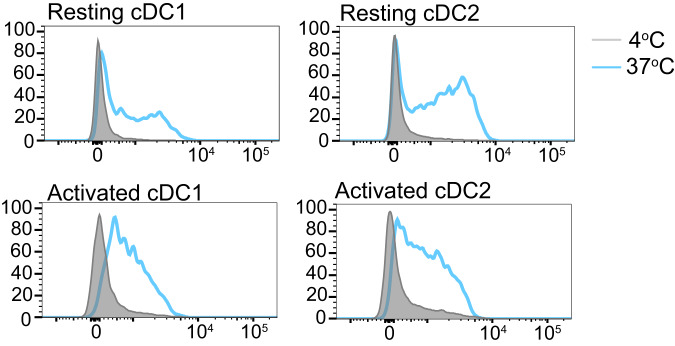
Primary DCs capture BPs in vitro. Primary DCs were isolated from the spleens of C57BL/6 mice and left at 4 °C (resting) or incubated with CpG at 37 °C (activated) overnight. DCs were then incubated with Nile-Red labelled BPs for four hours at 4 °C or 37 °C and then stained with CD11c and CD8 to identify cDC1 (CD11c^+^ CD8^+^) and cDC2 (CD11c^+^ CD8^−^) by flow cytometry. A representative of two independent experiments is shown.

**Fig. 4:**
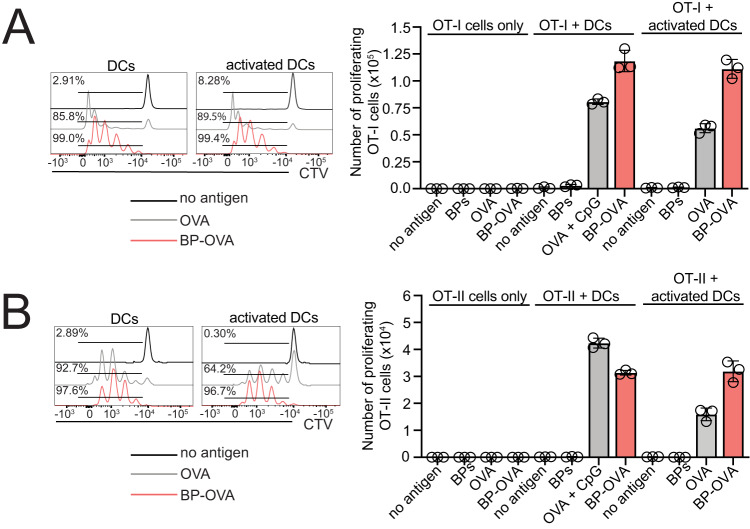
DCs present antigen associated with BP-OVA to CD8^+^ and CD4^+^ T cells in vitro. **A**, **B** 5 × 104 purified OT-I or OT-II were labelled with Cell Trace Violet (CTV) and incubated with 2.5 × 104 unstimulated DCs or DCs stimulated with CpG overnight. DCs were left untreated or incubated with 100 μg OVA, 100 μg OVA plus 5 μΜ CpG, 100 μg BP-OVA (278 ng OVA antigen) or 100 μg BP for 36 - 42 h. Division of Ly5.1^+^ TCRVα2^+^ CD8^+^ CTV-labelled OT-I and Ly5.1^+^ TCRVα2^+^ CD4^+^ OT-II T cells was assessed by flow cytometry. Histograms show proliferating OT-I or OT-II based on CTV dilution. One independent experiment was performed in triplicate. Bars represent mean ± SD.

To elucidate if primary DCs are capable of presenting antigen attached to BPs, in vitro antigen presentation assays with BP-OVA were performed. Primary DCs were purified from the spleen of C57BL/6 mice and assays performed with unstimulated DCs or DCs activated following overnight stimulation with TLR9 agonist, CpG. Both resting and activated cDC1 and cDC2 were capable of acquiring BPs, particularly when activated (Fig. 3). To test the capacity of DCs to process and present BP associated antigen, we examined their ability to present 100 μg BP-OVA. 100 μg refers to the mass of the BPs, which in this experiment equated to approximately 300 ng OVA antigen. 100 μg OVA protein either alone or in the presence of CpG were used as positive controls and 100 μg BPs (empty beads) were included as a negative control. OT-I, CD8^+^ T cells specific for H-2K^b^-OVA_257-264_ and OT-II, CD4^+^ T cells specific for IA^b^-OVA_323-332_ were purified and labelled with Cell Trace Violet (CTV) for analysis of antigen-specific T cell proliferation by flow cytometry. BP-OVA caused proliferation of OT-I cells, albeit at reduced division rates compared to OVA protein controls (Fig. 4A**)**. BP-OVA also promoted OT-II cell proliferation, similar to OVA protein controls (Fig. 4B). As expected, empty BPs without antigen did not elicit OT-I or OT-II cell division (Fig. 4B). This confirmed that both unstimulated and activated DCs could acquire antigen attached to BPs and were capable of processing it for presentation via both MHC I and MHC II antigen presentation pathways.

### Subcutaneous vaccination with BP-OVA induces CD4^+^ and CD8^+^ T cell responses in vivo

**Fig. 5:**
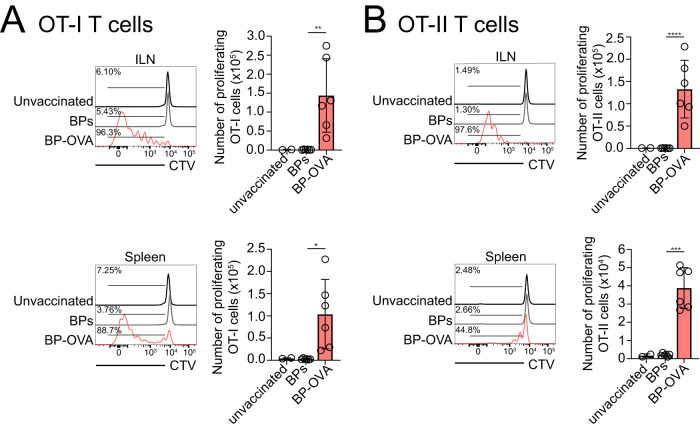
Subcutaneous vaccination with BP-OVA induces CD4^+^ and CD8^+^ T cell responses in vivo. 1 × 10^6^ purified OT-I (**A**) and OT-II (**B**) T cells were CTV labelled and injected intravenously into C57BL/6 mice. Mice were subcutaneously injected the following day with 5 mg BP or 5 mg BP-OVA (875 ng OVA antigen) and ILN and spleens harvested for analysis 60 - 64 h later. Division of CTV-labelled Ly5.1^+^ TCRVα2^+^ CD8^+^ OT-I and Ly5.1^+^ TCRVα2^+^ CD4^+^ OT-II T cells was assessed by flow cytometry. Representative histograms show proliferating OT-I or OT-II. Two independent experiments were performed in triplicate and each dot represents an individual mouse. Statistical analysis was performed using one-way ANOVA with Tukey’s multiple comparison test. Bars represent ±SD. *p < 0.05, **p < 0.01, ***p < 0.001, ****p < 0.0001.

Next, antigen-specific immunity to BP-OVA was tested in vivo. Mice were immunized subcutaneously with 5 mg BP-OVA. Twenty four hours later mice were adoptively transferred with Cell Trace Violet labelled OT-I and OT-II cells. Responses were examined in the ILN that drains the skin, and the spleen, 36 h later (gating strategy in Supplementary Fig. 2A). Following subcutaneous vaccination, both OT-I (Fig. 5A) and OT-II (Fig. 5B) cell division was detected in the ILN and spleen in response to BP-OVA, but not BP without antigen.

**Fig. 6:**
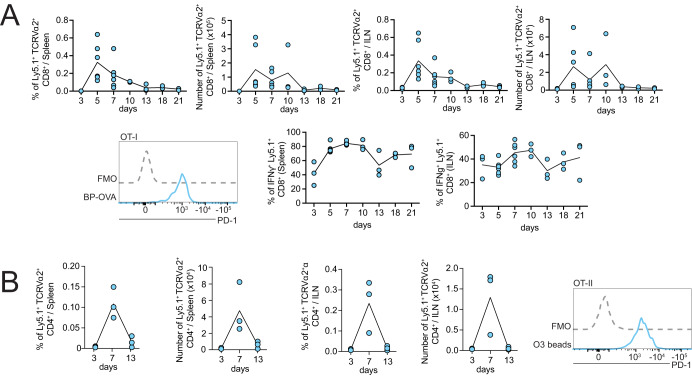
Kinetics of T cell responses following subcutaneous vaccination with BP-OVA. 10^4^ OT-I or OT-II cells were purified and injected to C57BL/6 one day prior to subcutaneous injection with 5 mg BP-OVA (2.825 μg OVA antigen). Organs were harvested from euthanised mice at 3, 5, 7, 10, 13, 18, and 21 days post-vaccination. **A** Frequency, number, PD-1 and IFNg expression by Ly5.1^+^ TCRVα2^+^ CD8^+^ OT-I in spleen and ILN. **B** Frequency, number, and PD1 expression by Ly5.1^+^ TCRVα2^+^ CD4^+^ OT-II cells in spleen and ILN. 1-2 independent experiments were performed with each dot representing an individual mouse. Bars represent ±SD. FMO is a background control “fluorescence minus one” stain, where the antibody specific for the marker examined is excluded from the staining panel.

To assess the kinetics of the T cell response to BP-OVA, mice were transferred with low numbers of OT-I and OT-II cells, immunised subcutaneously and T cell numbers and phenotypes examined after vaccination in the spleen and ILN up to 13 and 21 days, respectively, (gating strategy in Supplementary Fig. 2B). Both OT-I and OT-II responses were undetectable in either the spleen or ILN prior to day three. At both sites, expanded numbers of OT-I and OT-II T cells peaked at days five to seven before declining to very low numbers from day 13 (Fig. 6A, B). Expression of programmed cell death protein 1 (PD1), a marker of T cell activation, by OT-I and OT-II T cells in spleen was detected as early as day three post immunisation and waned by day 13. To measure effector T cell activity, spleen and ILN cells were isolated and restimulated in vitro with OVA_257-264_ peptide in the presence of Golgi Plug to block cytokine egress. Intracellular IFNγ staining showed that in response to BP-OVA, OT-I cells in the spleen and ILN generate IFNγ and maintain high levels of IFNγ production even when T cell numbers decline. In conclusion, BP-OVA elicited robust CD8^+^ and CD4^+^ T cell responses in vivo.

### BP-OVA vaccination elicits cytotoxic T lymphocyte killing in vivo

**Fig. 7:**
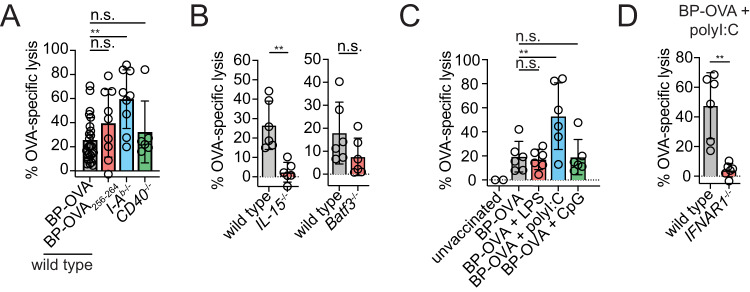
BP-OVA vaccination elicits cytotoxic T lymphocyte killing in vivo. **A** C57BL/6 wild type (WT) mice were subcutaneously injected with 5 mg BP-OVA or BP-OVA_257-264_ and *I-Ab*^−/−^ or *CD40*^−/−^ mice subcutaneously injected with 5 mg BP-OVA. **B** WT, *IL-15*^−/−^ and *Batf3*^−/−^ mice were subcutaneously injected with 5 mg BP-OVA. **C** WT mice were injected with 5 mg BP-OVA together with 1 μg LPS, 20 μg polyI:C or 20 nmol CpG. **D** WT mice and *IFNAR1*^−/−^ mice were subcutaneously injected with 5 mg of BP-OVA together with 20 μg polyI:C. **A**–**D** Six days following immunisation mice were injected intravenously with a 1:1 ratio of CTV^high^ OVA_257-264_^+^ and CTV^low^ unpulsed target cells and CTL activity in spleen measured 36 - 42 h later by flow cytometry. Data is pooled from 2 - 3 independent experiments with each symbol representing an individual mouse. Statistical analysis was performed using *t*-test and one-way ANOVA with Tukey’s multiple comparison test. Bars represent mean ± SD. *p < 0.05, **p < 0.01, ***p < 0.001, ****p < 0.0001, n.s. = not significant.

The ability of vaccination with BP-OVA to elicit cytotoxic T lymphocyte (CTL) immunity was examined. Responses were tested following subcutaneous administration of 5 mg BP-OVA containing OVA_257-264_ and OVA_323-332_ or 5 mg BP-OVA_257-264_ containing the OVA_257-264_ peptide alone. CTL activity was measured by transfer of CTV-labelled target cells pulsed or not, with the OVA_254-267_ peptide. Both BP-OVA and BP-OVA_257-264_ elicited approximately 20–30% killing activity against OVA_257-264_-pulsed target cells. Therefore, antigens displayed on BPs could elicit CTL killing, even in the absence of an antigen-specific MHC II peptide epitope. A contribution for CD4^+^ T helper cells, which for many vaccines is critical for effective CTL generation^16^ was examined using mice with MHC II *I-A*^*b*^ deletion^17^, lacking CD4^+^ T cells, and *CD40*^−/−^ mice deficient in CD40-CD40L signalling critical for CD4^+^ T cell help^18,19^. An anti-OVA CTL response was generated independent of CD4^+^ helper T cells or CD40 signalling. The increase in CTL killing in *I-A*^*b*^^−/−^ mice can be explained by the expanded numbers of CD8^+^ T cells in these mice^17^ (Fig. 7A). In response to both BP-OVA CTL killing was detected in spleen and BP-OVA also elicited an antigen-specific CTL response that was detectable in ILN with similar killing to that observed in the spleen (Supplementary Fig. 3). A role for IL-15, required for CD8^+^ T cell differentiation^20^, was examined using *IL-15*^−/−^ mice^21^ and indeed a role for IL-15 was important for CTL killing in response to BP-OVA. In addtion, the role of cDC1, the major cDC subset responsible for cross-presentation and anti-tumour immunity in mice^22^ in CTL killing in response to BP-OVA was determined. This was achieved using *Batf3*^−/−^ mice that lack Batf3, a transcription factor crucial for CD8^+^ and CD103^+^ cDC1 development^22^. In the absence of Batf3, CTL responses were reduced, but still detectable suggesting that cDC1, participate in, but are not critical for, CTL killing in response to BP-OVA (Fig. 7B). Next, we aimed to boost CTL killing in response to BP-OVA. To do this, we vaccinated mice with BP-OVA alone or in combination with the synthetic toll like receptor agonists CpG, LPS or polyinosinic:polycytidylic acid (poly I:C). Inclusion of LPS or CpG did not further enhance the CTL response to BP-OVA. In contrast, inclusion of poly I:C significantly increased CTL activity in response to BP-OVA in the spleen and ILN (Fig. 7C and Supplementary Fig. 3) by 2.7 and 2.8-fold, respectively. Poly I:C mimics the presence of double stranded RNA and stimulates type I interferon (IFN) production. Therefore, we hypothesised type I IFN enhances CTL killing in response to BP-OVA. To investigate this, CTL killing was investigated in *IFNAR1*^−/−^ mice where the type I IFN receptor is absent and cells are incapable of signalling type I IFN-dependent immune responses^23^. In the absence of *IFNAR1*, BP-OVA, in the presence of poly I:C, failed to generate CTL killers, suggesting that T cell responses to BP-OVA are indeed amplified by type I IFN (Fig. 7D).

### BP-OVA vaccination elicits anti-tumour immunity

**Fig. 8:**
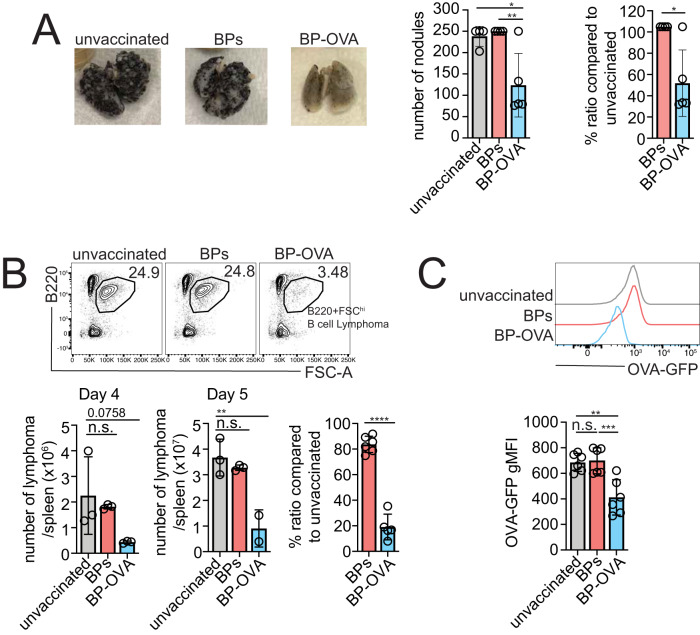
BP-OVA vaccination elicits anti-tumour immunity. **A** C57BL/6 mice were subcutaneously injected with 5 mg BP-OVA (2.8 μg OVA antigen) seven days prior to intravenous inoculation B16-OVA melanoma. Lungs were assessed for tumour nodules after 18 days following tumour inoculation. Pictures represent the lungs of mice in each immunisation group. **B**, **C** C57BL/6 mice were subcutaneously injected with 5 mg (2.8 μg OVA antigen) BP-OVA five days prior to intravenous inoculation with Eμ-myc-GFP-OVA lymphoma. Spleens were harvested four or five days after tumour inoculation and analysed by flow cytometry. **B** Dot plots show representative Eμ-myc lymphoma cells (B220^+^ FSC^hi^), total number of lymphoma cells and **C** GFP expression following Eμ-myc-GFP-OVA inoculation. Data is pooled from up to two independent experiments with each symbol representing an individual mouse. Statistical analysis was performed using t-test and one-way ANOVA with Tukey’s multiple comparison test. Bars represent ±SD. n.s. = not significant; *p < 0.05; **p < 0.01; ****p < 0.0001.

The utility of BP-OVA as a vaccine for anti-tumour immunity was measured in two mouse models of tumour immunotherapy, melanoma and B cell lymphoma. B16-OVA cells are B16F10 melanoma cells that express membrane-bound OVA^24^. B16-OVA cells are inoculated intravenously with tumours developing in lungs. C57BL/6 mice were vaccinated subcutaneously with BP-OVA seven days prior to tumour inoculation. Lungs were harvested after 18 days and tumour nodules examined. BP-OVA successfully induced an anti-tumour response against intravenous B16-OVA with significantly lower numbers and percentages of tumour nodules in the lungs compared to BPs without OVA antigen or unvaccinated mice (Fig. 8A). Next, anti-tumour immunity elicited by BP-OVA was examined in an Eμ-myc B cell lymphoma model. Eμ-myc-GFP-OVA are Eμ-myc cells engineered to express OVA and GFP (for detection by flow cytometry). The lymphoma cells are inoculated intravenously and home to the spleen. C57BL/6 mice were vaccinated subcutaneously with BP-OVA four days prior to inoculation of Eμ-myc-GFP-OVA lymphoma. Spleens were harvested four or five days after tumour inoculation for analysis by flow cytometry. A significant decrease in lymphoma burden was detected in BP-OVA vaccinated mice, compared to unvaccinated mice and mice immunized with empty BPs (Fig. 8B). Loss of GFP was also observed after BP-OVA vaccination indicating that a portion of the Eμ-myc-GFP-OVA cells have undergone immune editing with these cells no longer express OVA. Therefore, BP-OVA is capable of eliciting anti-tumour immunity, even in the absence of adjuvant.

## Discussion

Here, we demonstrate robust immunogenic potential of BPs as a carrier of MHC I and MHC II- restricted antigenic epitopes. BPs themselves were weakly immunogenic and capable of eliciting cytokine production. When coated with epitopes, BPs elicited DC-mediated MHC I and MHC II antigen presentation, CD4^+^ and CD8^+^ T cell priming resulting in differentiation into CTLs capable of mediating effective anti-tumour immunity.

BP vaccination is weakly immunogenic, with the induction of low levels of cytokine production, including by DCs and low levels of evident DC activation based on their cell surface phenotype. Despite this apparent lack of overt immunogenicity, subcutaneous immunisation with epitope-coated BP-OVA elicited CD8^+^ T cell responses even in the absence of adjuvant, elicited OVA-specific IFNγ-expressing CD8^+^ T cells, OVA-specific CD4^+^ T cell responses and importantly, CTL killers. Moreover, CTL immunity elicited by BP-OVA likely contributes to the efficacy of BP-OVA vaccination promoting effective antigen-specific tumour eradication in models of melanoma and B cell lymphoma. To examine the T cell parameters responsible for the efficacy of BP-OVA vaccination, a panel of gene deleted mice were examined. First, we examined IL-15, critical for CD8^+^ T cell effector function^20^ and indeed, CTL responses to BP-OVA were significantly impaired in *IL-15*-deficient mice. Despite inducing a significant expansion of anti-OVA CD4^+^ T cells, surprisingly, BP-OVA could generate CTL killing in the absence of CD4^+^ T cell help. The ability of BP-OVA to induce CTL was unperturbed by a lack of MHC II and/or CD40 signalling. CD4^+^ T cell help is critical for robust CTL immunity to many immunogens, promoting CD8^+^ T cell expansion, their differentiation into killers and survival^16,18^. Accordingly, engineering MHC II epitopes into vaccine platforms is considered an effective strategy to boost vaccine induced CTL responses^25^, however in this case immunisation with BP-OVA_257-264_ alone elicited a CTL response similar in magnitude, indicating in this setting inclusion of an antigen- MHC II helper T cell epitope does not impact or boost CTL outcomes. One signal that enables CD4^+^ T cell help to be bypassed in CTL induction is type I IFN^26^. Notably, we observed that in the absence of IFNAR, CTL responses to BP-OVA were reduced, suggesting type I IFN is indeed elicited by BP vaccination. Poly I:C signals via TLR3 to promote type I IFN production, and its coadministration with BP-OVA effectively boosted CTL killing. This was not the case for coadministration of CpG or LPS. TLR3 is highly expressed by DCs, in particular CD8^+^ and CD103^+^ cDC1^27,28^, macrophages, fibroblasts and epithelial cells^29^. To test, the contribution of CD8^+^ and CD103^+^ cDC1 to anti-BP-OVA CTL immunity, *Batf3*-deficient mice were vaccinated. Batf3 is a basic leucine zipper ATF-like (BATF) transcription factor and critical for cDC1 development. *Batf3*-deficient mice^22^ lack CD103^+^ DCs and a proportion of CD8^+^ splenic DCs. CD103^+^ DCs reside in tissues, including the skin, where they capture antigen and migrate to the draining lymph node^30^ and therefore were considered likely participants in the immune response to BP-OVA. Surprisingly, *Batf3*-deficient mice remained capable of generating CTL killers in response to BP-OVA, albeit at reduced levels. These mice are not completely devoid of cross-presenting antigen presenting cells, however as in the absence of *Batf3* pre-cDC1 develop normally with these cells capable of cross-presentation after immunization with anti-Clec9a-OVA^31^. Other antigen presenting cell subsets may also contribute to this response namely cDC2 and/or CD169^+^ macrophages for which there is also evidence of a capacity to cross-present^32,33^. Finally, it is possible that B cell responses and antibody production are contributing to tumour elimination elicited by BP vaccination, particularly given the efficacy by which this mode of vaccination elicits humoral immunity^7–9^.

Given the size of BPs used in this study (>500 nm), BP-OVA is most likely captured via phagocytosis^34^. Our analysis shows BP associated antigen, in this case OVA epitopes, is subsequently processed and cross-presented by MHC I and presented by MHC II. Phagocytosis is a major internalisation route that enables antigen to access the cross-presentation pathway^35^. MHC I cross-presentation of BP-OVA_257-264_ likely occurs following uptake of BPs into the early endosomes, endosomal escape and MHC I loading in the endoplasmic reticulum. It is unclear if BPs escape endosomes or only the associated polypeptides. The potential of BPs and/or their associated cargo to access the cytosol is an attractive property given that trapping of nanomaterials in endosomal compartments is a major rate limiting step in their use as therapeutics^36^. An alternative is that BP-associated antigen is loaded into MHC I localised in endosomes. Regardless, the ability of BPs to promote MHC I cross-presentation of antigen, in addition to providing antigen for MHC II loading, significantly expands their utility as vaccines given that access to MHC I cross-presentation pathway enables them to elicit CTL immunity. In summary, our study shows that BPs can be precision-engineered to serve as epitope delivery platforms that following single dose immunisation can effectively elicit T cell responses, including CTLs, and can be used to effectivity elicit immune-mediated tumour killing capacity.

## Methods

### Biopolymer synthesis

ClearColi™ (Lucigen, Middleton, WI), an endotoxin-free mutant of *Escherichia coli* BL21(DE3), harboring pMCS69^14^ was transformed with cloned pET-14b-PhaC (Supplementary Table 1) containing DNA encoding SIINFEKL and ISQAVHAAHAEINEAGR to synthesize BP-OVA as well as empty BPs (control). Cells were cultivated in Luria–Bertani (LB) media containing 100 mg/L ampicillin and 50 mg/L chloramphenicol supplemented with 1% (w/v) glucose at 37 °C and 200 rpm for 20 h. Overnight cultures were seeded into 500 mL of LB media at an OD600 of 0.05 and incubated at 37 °C and 200 rpm until an OD600 of 0.6–0.8 was achieved. BP production was induced by adding 1 mM isopropyl β-D-1-thiogalactopyranoside and further growth at 25 °C for 48 h. Cells were harvested and disrupted releasing BPs which were subjected to a series of washes resulting in purified BPs as previously described^7,9–13^. BP suspensions were sterilized and stored in 10 mM Tris buffer pH7.5 containing 20% (v/v) ethanol at 4 °C. Sterility was confirmed by the absence of bacterial colonies after streaking the bead suspension on solid media and incubation of the particle-containing agar plate at 37 °C for 48 h.

### Staining of BPs

Purified BPs were stained with lipophilic dye Nile red (Thermo Fisher Scientific, Australia). Briefly, 100 μL of a bead suspension (0.2 g/mL in 10 mM Tris pH 7.5) was mixed thoroughly with 10 μL of Nile red (0.2 mg/mL in dimethyl sulfoxide). The suspension was washed three times with 10 mM Tris buffer pH7.5 by centrifugation (8000 × *g*, 4 °C, 5 min) and stored in the dark prior to analysis.

### Biopolymer particle characterization

Protein profiles of BPs were analysed by sodium dodecyl sulfate polyacrylamide gel electrophoresis (SDS-PAGE) using 10% Bis-Tris gels mounted in a Bio-Rad XCell 3 system (Bio-Rad Laboratories, CA) with 3-(*N*-morpholino)propanesulfonic acid (MOPS) buffer and GangNam^TM^-STAIN Prestained Protein Ladder (iNtRON Biotechnology, South Korea). Densitometry analyses of SDS-PAGE gels were used for protein quantification. Sequences of fusion proteins expressed by BPs were identified based on peptide mass fingerprinting by first excising the target protein bands from the SDS-PAGE gel and mixing with trypsin gold (Promega Corporation, WI). After desalting and filtration, tryptic peptides were subjected to a liquid chromatography coupled with tandem mass spectrometry (LC-MS/MS) (Sciex, MA). The content of poly(3-hydroxybutyrate) (PHB) within the beads as expressed in PHB weight (mg) per wet bead weight (g) was measured using a high-performance liquid chromatography system (Agilent Technologies, CA) after acid digestion of freeze-dried beads. Size distribution and zeta potentials of BPs resuspended in 10 mM Tris buffer pH 7.5 were determined based on dynamic light scattering (DLS) using Malvern Zetasizer Nano ZS (Malvern Instrument, UK) at 25 °C with a scattering angle of 173°. Prior to size measurements, BP suspensions were diluted to avoid multiple scattering effects. Morphologies of BPs when encapsulated in cells as well as after purification were visualized by transmission electron microscope (TEM) using a Hitachi HT7700 TEM (Hitachi High-Tech Corporation, Japan) operated at 80 kV. Prior to imaging, samples were fixed with glutaraldehyde and OsO_4_, then dehydrated in a graded ethanol series, followed by infiltration with an epoxy resin. After incubation at 60 °C for 48 h, the BP-embedded resin was sliced into 70 nm sections and stained using a double contrasting technique by sequentially adding 5% uranyl acetate in ethanol and Reynolds lead citrate solution^37^ for TEM. All blots and gels were processed in parallel and were derived from the same experiments.

### Mice

C57BL/6, OT-I/Ly5.1^38^, OT-II/Ly5.1^39^, I-A^b^^−/−^^17^, *IFNAR1*^−/−^^40^ were bred maintained in pathogen-free environment at the Melbourne Bioresources Platform at Bio21 Molecular Science and Biotechnology Institute. *Batf3*^−/−^^22^, *CD40*^−/−^^19^ and *IL-15*^−/−^^21^ mice were bred in Peter Doherty Institute (PDI) animal facility. Mice used were between 6–12 weeks old. Experimental procedures were approved by Animal Ethics Committee of the University of Melbourne (1714375 and 20150). The experimental endpoints varies from day 3 to day 21 and the mice were euthanised via CO_2_ asphyxiation in appropriate CO_2_ chambers. The spleens and/or lymph nodes were harvested for analysis by flow cytometry.

### MuTuDC cell line

MuTu dendritic cell line was provided by Hans Acha-Orbea (University of Lausanne, Switzerland)^41^. MuTu was cultured in Iscove’s Modified Dulbecco Medium-Glutamax (IMDM, Thermo Fischer) supplemented with 10% fetal bovine serum (FBS, Gibco), 100 μM β-mercaptoethanol, 100 IU/mL penicillin and 100 μg/ml streptomycin (37 °C, 10% CO_2_). To activate MuTu cells, 2 × 10^6^ cells were incubated in 0.5 µM CpG type B 1668 (Geneworks) overnight (14 - 16 h). Cells were stained with mAbs specific for MHC II (M5/114.15.2, AF700, 1:400, 107622) and CD86 (GL1, APC, 1:400, 105012) (Biolegend) for flow cytometry analysis using LSR Fortessa (BD Bioscience) and analyzed on FlowJo software (Tree Star).

### Immune cell analysis

C57BL/6 mice were injected with BPs subcutaneously and their inguinal lymph nodes (ILNs) harvested 16 h post-vaccination. For DCs subset analysis, primary DCs from ILNs were purified as previously described^42^. DCs were stained with mAbs (Biolegend) specific for: CD11c (N418, BV510, 1:200, 117338), MHC II (M5/114.15.2, AF700, 1:400, 107622), CD8 (53-6.7, BV421, 1:200, 100738), Sirpα (P84, FITC, 1:200, 144006), CD103 (2E7, BV605, 1:400, 121433) and Ep-CAM (G8.8, APC-Cy7, 1:400, 118217). For innate immune cells analysis, a single cell suspension was obtained by pushing ILNs through 40 μm sieve (Corning). Cells were stained for mAbs (Walter and Eliza Hall Antibody Facility, Biolegend or BD) specific for: CD3 (KT3-1.1, BV510, 1/100, 740147), CD4 (GK1.5, BV605, 1:200, 100451), CD8 (53-6.7, BV510, 1/400, 100752), CD11c (N418, BV510, 1:200, 117338), B220 (RA3-6B2, B510, 1:100, 103247), Ly6G (GR-1, FITC, 1:100, WEHI), NK1.1 (PK136, PE, 1:600, 553165), Ly6C (5075-3.6, APC-Cy7, 1:800, 128026), MHC II (M5/114.15.2, AF700, 1:400, 107622) and F4/80 (F4/80, A647, 1:600, WEHI). Migratory DCs were defined as MHC II^hi^ CD11c^int^ cells and further divided into: CD103^+^ dermal DCs, CD103^-^ dermal DCs, and Langerhans cells (EpCAM^+^). Resident DCs were defined as MHC II^int^ CD11c^hi^ cDC1 (CD8^+^) and cDC2 (Sirpα^+^). For innate immune cells, CD4, CD3, CD8, B220 and CD11c positive cells were excluded in a “dump” channel and cells gated on CD11b^+^. Neutrophils were defined as Ly6G^+^ and NK cells as Ly6G^-^ NK1.1^+^. After gating on Ly6G^-^ NK1.1^-^, eosinophils were defined as SSC-H^int^ Ly6C^-^, Ly6C^+^ monocytes as SSC-H^lo^ Ly6C^+^, Ly6C^-^ and macrophages as SSC-H^lo^ Ly6C^−^ F4/80^+^. Counting beads (Accucheck, Thermo Fischer) were added to allow quantification of immune cells. Analysis was performed using LSR Fortessa (BD Biosience).

### Cytometric bead array (CBA) assay

Splenic DCs were purified as previously described^43^. DCs were incubated for 24 h with 0.5 µM CpG Type B 1688 (Geneworks), 0.1 µg/ml lipopolysaccharide (LPS, Sigma) or 100 µg PBs. Supernatants were removed for analysis. C57BL/6 mice were injected with BPs subcutaneously and cardiac blood was collected and centrifuged for serum collection. Cytokines were measured using BD cytometric bead array (CBA) Mouse Inflammation Kit (BD Biosciences) as per the manufacturer’s instructions. Capture beads were specific for IL-6, IL-10, IL-12p70, MCP-1, IFNγ and TNFα. Statistical analysis was performed using GraphPad Prism 9 software.

### Enrichment of OT-I and OT-II T cells

Total LNs were harvested from OT-I × Ly5.1 and OT-II × Ly5.1 mice. Cells were stained with rat anti-mouse mAbs (WEHI) specific for: CD11b (M1/70), F4/80 (F4/80), red blood cells (TER119), Ly6G/-Ly6C (RB68C5), MHC II (M5/114), CD45R (RA3-6B2) and CD4 (GK1.5) for OT-I cells or CD8α (53-6.7) for OT-II cells followed by anti-rat IgG coupled with magnetic beads (Bio-Rad) to deplete and remove labelled cells. Enriched OT-I and OT-II cells were washed with phosphate buffered saline (PBS, MPU) supplemented with 0.1% bovine serum albumin (BSA). T cells were incubated with 5 µM Cell Trace Violet (CTV, Thermo Fischer) for 20 min at 37 °C in PBS 0.1% BSA. Cells were washed twice in KDS-RPMI 2% FBS.

### In vitro uptake assay

Primary splenic DCs were purified as previously described^43^. Activated primary DCs were incubated for 14–16 h in the presence of 0.5 µM CpG Type B 1688 (Geneworks). DCs were incubated with BPs labelled with Nile Red as previously described^44^ for 4 h at 4 °C and 37 °C and stained with CD11c (N418, FITC, 1:800, WEHI) and CD8 (53-6.7, BV421, 1:200, 100738, Biolegend) for flow cytometry analysis.

### In vitro antigen presentation assay

Primary splenic DCs were purified as previously described^43^. Activated primary DCs were incubated for 14–16 h in the presence of 0.5 µM CpG Type B 1688 (Geneworks). Unstimulated DCs were pulsed with 100 μg OVA (Worthington) together with 5 µM CpG, and activated splenic DCs pulsed with 100 μg OVA for 45 minutes at 37 °C. Excess OVA was removed by washing. Both unstimulated and activated DCs were pulsed with BP or BP-OVA beads that remained in culture. 5 × 104 CTV-labelled purified OT-I or OT-II T cells were cultured in vitro with unstimulated or activated DCs in RPMI 1640 supplemented with 10% FBS, 100 IU/mL penicillin (MPU) and 100 μg/ml streptomycin (MPU). Cells were incubated for 60 - 64 h (37 °C, 10% CO_2_). T cells were stained with mAbs specific for: CD4 (GK1.5, FITC, 1:500, 100406), CD8 (53-6.7, FITC, 1:400, WEHI), TCRVα2 (B20.1, APC, 1:800, WEHI) and Ly5.1 (A20.1, PE-Cy7, 1:200, 110730) (Biolegend). T cell proliferation was analyzed using flow cytometry and CTV dilution. Counting beads (Accucheck, Thermo Fischer) were included to enable determination of the number of proliferating T cells.

### In vivo antigen presentation assay

OT-I and OT-II cells were purified as described. C57BL/6 mice were adoptively transferred intravenously with CTV-labelled 1 × 10^6^ OT-I or OT-II cells. To ensure accurate quantification of T cell proliferation in vivo, 1 × 10^6^ CFSE-labelled splenocytes were also transferred. After 24 h, mice were subcutaneously injected with 5 mg BP or BP-OVA. After 60 - 64 h, spleens and ILNs were harvested. Splenocytes were treated with red blood cell removal buffer (RCRB, MPU) and single cell suspensions stained with mAbs specific for: CD4 (GK1.5, FITC, 1:500, 100406), CD8 (53-6.7, FITC, 1:400, WEHI), TCRVα2 (B20.1, APC, 1:800, WEHI) and Ly5.1 (A20.1, PE-Cy7, 1:200, 110730) (Biolegend). Dividing OT-I and OT-II were identified by flow cytometry. Counting beads (Accucheck, Thermo Fischer) were used to enable determination of the number of proliferating T cells.

### T cells kinetics in vivo

1 × 10^4^ purified OT-I or OT-II cells were injected intravenously into C57BL/6 mice. One day later, mice were vaccinated with 5 mg BP-OVA beads subcutaneously. Mice were euthanized 3, 5, 7, 10, 13 or 21 days post-vaccination and their spleens and ILNs harvested. Single cell suspensions were generated and splenocytes treated with RCRB. Cells were stained with mAbs (Biolegend or WEHI Antibody Facility) specific for: TCRVα2 (B20.1, FITC, 1:800, WEHI), Ly5.1 (A20, PE-Cy7, 1:200, 110730), PD-1 (29 F.1A12, APC, 1:200, 135210), CD8 (53-6.7, BV421, 1:200, 100738) for OT-I or CD4 (GK1.5, PE, 1:400, 100408), TCRVα2 (B20.1, FITC, 1:800, WEHI), Ly5.1 (A20.1, PE-Cy7, 1:200, 110730), PD-1 (29 F.1A12, BV421, 1:100, 135217) for OT-II. Counting beads (Accucheck, Thermo Fischer) were used to enable quantification of T cells. For IFNγ production, OT-I were stimulated with OVA_257-264_ in the presence of BD Golgi Plug^TM^ (5 h; 37 °C). Cells were stained for CD8 (53-6.7, BV421, 1:200, 100738), TCRVα2 (B20.1, FITC, 1:800, WEHI) and Ly5.1 (A20.1, PE-Cy7, 1:200, 110730) prior to permeabilization and fixation using BD Cytofix/Cytoperm Kit. Permeabilised cells were stained for IFNγ (XMG1.2, PE, 1:200, 554412, BD) and analysed by flow cytometry. IFNγ analysis was only conducted at time points where Ly5.1^+^ CD8^+^ TCRVα2^+^ cells represented more than 0.1% of total cells.

### Cytotoxic T cell assay

Mice were injected with 5 mg of BP-OVA beads subcutaneously, in the presence of absence of 1 μg of CpG type B 1668 (Geneworks), 20 μg of polyI:C (InVivoGen) or 1 μg of LPS (Sigma). After six days, splenocytes were isolated from naïve C57B/L6 mice and prepared as target cells. Half the splenocyte suspension was pulsed (40 min at 37 °C) with 30 ng OVA_257-264_ peptide (Worthington) and the other half left untreated. Peptide-pulsed cells and non-pulsed cells were stained (20 min at 37 °C) with 5 µM and 0.5 µM CTV (Thermo Fischer), respectively. Excess dye was removed by washing. CTV-high OVA_257-264_-pulsed and CTV-low unpulsed cells were pooled at a ratio of 1:1 and 10^7^ cells injected intravenously. After 36 - 42 h, splenocytes were isolated to analyze CTL activity. Statistical analysis was performed using GraphPad Prism 9 software.

Calculation of % OVA-specific lysis is determined by:

$$\begin{array}{l}{{R}}=( \% {{\rm{CTV}}}^{{\rm{low}}}/ \% \,{{\rm{CTV}}}^{{\rm{high}}}) \% {\rm{OVA}}-{\rm{specific}}\; {\rm{lysis}}\\\quad=\,\left[\right.1-({{{r}}}_{{\rm{unprimed}}}/{{{r}}}_{{\rm{primed}}})\,{{\times}}\,100\end{array}$$

### Tumour assays

C57BL/6 mice were vaccinated with 5 mg BP-OVA seven days prior to intravenous incoculation with 2.5 × 10^5^ B16-OVA melanoma cells^45^ or five days prior to intravenous innoculation with 1 × 10^6^ Eμ-myc-OVA-GFP lymphoma cells^46^. 18 days after B16-OVA inoculation, lungs were harvested and preserved in Fekete’s solution^47^ (for 1 l: 580 ml of 95% ethanol, 200 ml of H_2_O, 80 ml of 37% formaldehyde and 40 ml glacial acetic acid). Tumour nodules were scored, with the maximum number of tumours detected set at 250. For Eμ-myc-OVA-GFP lymphoma, spleens were harvested to obtain single cell suspension. Cells were stained with B220 (RA3-6B2, PE, 1:100, 103208, Biolegend) to allow quantification of Eμ-myc-OVA-GFP lymphoma in spleen at day four and five following tumour innoculation. Statistical analysis was performed using GraphPad Prism 9 software.

### Reporting summary

Further information on research design is available in the Nature Research Reporting Summary linked to this article.

### Supplementary information

Reporting Summary

Supplemental Material

## Data Availability

All data required conclusion are available in this paper and/or supplementary paper, except for DNA and amino acid sequences for BP will only be made available upon reasonable request subject to a confidentiality agreement. Materials are available upon request subject to a Material Transfer Agreement.
