# Supplementary material for: In vivo assembly of epitope-coated biopolymer particles that induce anti-tumor responses
Source: NPJ Vaccines. 2024 Jan 23;9:18. doi: 10.1038/s41541-023-00787-8 (PMC10805745; doi:10.1038/s41541-023-00787-8)
Supplement: Supplementary file 1 — Reporting Summary [file 41541_2023_787_MOESM1_ESM.pdf]

nature portfolio

Corresponding author(s): Justine Mintern

Last updated by author(s): Sep 17, 2023

Reporting Summary

Nature Portfolio wishes to improve the reproducibility of the work that we publish. This form provides structure for consistency and transparency in reporting. For further information on Nature Portfolio policies, see our Editorial Policies and the Editorial Policy Checklist.

Statistics

For all statistical analyses, confirm that the following items are present in the figure legend, table legend, main text, or Methods section.

n/a	Confirmed	
☐	☒	The exact sample size (n) for each experimental group/condition, given as a discrete number and unit of measurement
☐	☒	A statement on whether measurements were taken from distinct samples or whether the same sample was measured repeatedly
☐	☒	The statistical test(s) used AND whether they are one- or two-sided<br>*Only common tests should be described solely by name; describe more complex techniques in the Methods section.*
☐	☒	A description of all covariates tested
☐	☒	A description of any assumptions or corrections, such as tests of normality and adjustment for multiple comparisons
☐	☒	A full description of the statistical parameters including central tendency (e.g. means) or other basic estimates (e.g. regression coefficient) AND variation (e.g. standard deviation) or associated estimates of uncertainty (e.g. confidence intervals)
☐	☒	For null hypothesis testing, the test statistic (e.g. F, t, r) with confidence intervals, effect sizes, degrees of freedom and P value noted<br>*Give P values as exact values whenever suitable.*
☒	☐	For Bayesian analysis, information on the choice of priors and Markov chain Monte Carlo settings
☒	☐	For hierarchical and complex designs, identification of the appropriate level for tests and full reporting of outcomes
☒	☐	Estimates of effect sizes (e.g. Cohen's d, Pearson's r), indicating how they were calculated

Our web collection on statistics for biologists contains articles on many of the points above.

Software and code

Policy information about availability of computer code

Data collection	no software was used
Data analysis	FlowJo was used to analyse flow cytometry data.

For manuscripts utilizing custom algorithms or software that are central to the research but not yet described in published literature, software must be made available to editors and reviewers. We strongly encourage code deposition in a community repository (e.g. GitHub). See the Nature Portfolio guidelines for submitting code & software for further information.

Data

Policy information about availability of data

All manuscripts must include a data availability statement. This statement should provide the following information, where applicable:

- Accession codes, unique identifiers, or web links for publicly available datasets
- A description of any restrictions on data availability
- For clinical datasets or third party data, please ensure that the statement adheres to our policy

“No datasets were generated or analysed during the current study”

Research involving human participants, their data, or biological material

Policy information about studies with human participants or human data. See also policy information about sex, gender (identity/presentation), and sexual orientation and race, ethnicity and racism.

Reporting on sex and gender	*Use the terms sex (biological attribute) and gender (shaped by social and cultural circumstances) carefully in order to avoid confusing both terms. Indicate if findings apply to only one sex or gender; describe whether sex and gender were considered in study design; whether sex and/or gender was determined based on self-reporting or assigned and methods used. Provide in the source data disaggregated sex and gender data, where this information has been collected, and if consent has been obtained for sharing of individual-level data; provide overall numbers in this Reporting Summary. Please state if this information has not been collected. Report sex- and gender-based analyses where performed, justify reasons for lack of sex- and gender-based analysis.*
Reporting on race, ethnicity, or other socially relevant groupings	*Please specify the socially constructed or socially relevant categorization variable(s) used in your manuscript and explain why they were used. Please note that such variables should not be used as proxies for other socially constructed/relevant variables (for example, race or ethnicity should not be used as a proxy for socioeconomic status). Provide clear definitions of the relevant terms used, how they were provided (by the participants/respondents, the researchers, or third parties), and the method(s) used to classify people into the different categories (e.g. self-report, census or administrative data, social media data, etc.) Please provide details about how you controlled for confounding variables in your analyses.*
Population characteristics	*Describe the covariate-relevant population characteristics of the human research participants (e.g. age, genotypic information, past and current diagnosis and treatment categories). If you filled out the behavioural & social sciences study design questions and have nothing to add here, write "See above."*
Recruitment	*Describe how participants were recruited. Outline any potential self-selection bias or other biases that may be present and how these are likely to impact results.*
Ethics oversight	*Identify the organization(s) that approved the study protocol.*

Note that full information on the approval of the study protocol must also be provided in the manuscript.

Field-specific reporting

Please select the one below that is the best fit for your research. If you are not sure, read the appropriate sections before making your selection.

☒ Life sciences ☐ Behavioural & social sciences ☐ Ecological, evolutionary & environmental sciences

For a reference copy of the document with all sections, see nature.com/documents/nr-reporting-summary-flat.pdf

Life sciences study design

All studies must disclose on these points even when the disclosure is negative.

Sample size	Sample size was determined to ensure statistical power.
Data exclusions	No data was excluded
Replication	Experiments were repeated using independent biological samples.
Randomization	Mice were randomly assigned to experimental or control groups.
Blinding	Blinding was not relevant for this study as there were no subjective measurements being made.

Reporting for specific materials, systems and methods

We require information from authors about some types of materials, experimental systems and methods used in many studies. Here, indicate whether each material, system or method listed is relevant to your study. If you are not sure if a list item applies to your research, read the appropriate section before selecting a response.

Materials & experimental systems

n/a	Involved in the study
☐	☒ Antibodies
☐	☒ Eukaryotic cell lines
☒	☐ Palaeontology and archaeology
☐	☒ Animals and other organisms
☒	☐ Clinical data
☒	☐ Dual use research of concern
☒	☐ Plants

Methods

n/a	Involved in the study
☒	☐ ChIP-seq
☐	☒ Flow cytometry
☒	☐ MRI-based neuroimaging

Antibodies

Antibodies used

CD3 (KT3-1.1), CD4 (GK1.5), CD8 (53-6.7), CD11c (N418), B220 (RA3-6B2), Ly6G (GR-1), NK1.1 (PK136), Ly6C (5075-3.6), MHC II (M5/114.15.2) and F4/80 (F4/80). Antibodies purchased from Biolegend or generated by Walter and Eliza Hall Institute.

Validation

Antibodies were validated based on the expected staining profiles elicited following use for flow cytometery.

Eukaryotic cell lines

Policy information about cell lines and Sex and Gender in Research

Cell line source(s)

MuTu dendritic cell line, sourced from Hans Acha Orbea, Switzerland; B16-OVA melanoma cell line sourced from A/Prof Irene Caminschi, Monash University.

Authentication

MuTu DCs were authenicated based on flow cytometry analysis, B16-OVA were authenticated by growth patterns in mice and in presence of anti-tumour T cell response.

Mycoplasma contamination

All cell lines were tested for mycoplasma.

Commonly misidentified lines
(See ICLAC register)

Name any commonly misidentified cell lines used in the study and provide a rationale for their use.

Animals and other research organisms

Policy information about studies involving animals; ARRIVE guidelines recommended for reporting animal research, and Sex and Gender in Research

Laboratory animals

C57BL/6, OT-I/Ly5.116, OT-II/Ly5.117, I-Ab-/-18, IFNAR1-/-19 were bred maintained in pathogen-free environment at the Melbourne Bioresources Platform at Bio21 Molecular Science and Biotechnology Institute. Batf3-/-20, CD40-/-21 and IL-15-/-22 mice were bred in Peter Doherty Institute (PDI) animal facility. Mice used were between 6 - 12 weeks old.

Wild animals

Provide details on animals observed in or captured in the field; report species and age where possible. Describe how animals were caught and transported and what happened to captive animals after the study (if killed, explain why and describe method; if released, say where and when) OR state that the study did not involve wild animals.

Reporting on sex

Indicate if findings apply to only one sex; describe whether sex was considered in study design, methods used for assigning sex. Provide data disaggregated for sex where this information has been collected in the source data as appropriate; provide overall numbers in this Reporting Summary. Please state if this information has not been collected. Report sex-based analyses where performed, justify reasons for lack of sex-based analysis.

Field-collected samples

For laboratory work with field-collected samples, describe all relevant parameters such as housing, maintenance, temperature, photoperiod and end-of-experiment protocol OR state that the study did not involve samples collected from the field.

Ethics oversight

Experimental procedures were approved by Animal Ethics Committee of the University of Melbourne (1714375 and 20150).

Note that full information on the approval of the study protocol must also be provided in the manuscript.

Flow Cytometry

Plots

Confirm that:

☒ The axis labels state the marker and fluorochrome used (e.g. CD4-FITC).

☒ The axis scales are clearly visible. Include numbers along axes only for bottom left plot of group (a 'group' is an analysis of identical markers).

☒ All plots are contour plots with outliers or pseudocolor plots.

☒ A numerical value for number of cells or percentage (with statistics) is provided.

Methodology

Sample preparation	For DCs subset analysis, primary DCs from ILNs were purified as previously described24. DCs were stained with mAbs (Biolegend) specific for: CD11c (N418), MHC II (M5/114.15.2), CD8 (53-6.7), Sirp⍰ (P84), CD103 (2E7) and EpCAM (G8.8). For innate immune cells analysis, a single cell suspension was obtained by pushing ILNs through 40⍰m sieve (Corning). Cells were stained for mAbs (Walter and Eliza Hall Antibody Facility or Biolegend) specific for: CD3 (KT3-1.1), CD4 (GK1.5), CD8 (53-6.7), CD11c (N418), B220 (RA3-6B2), Ly6G (GR-1), NK1.1 (PK136), Ly6C (5075-3.6), MHC II (M5/114.15.2) and F4/80 (F4/80). Migratory DCs were defined as MHC IIhi CD11cint cells and further divided into: CD103+ dermal DCs, CD103- dermal DCs, and Langerhans cells (EpCAM+). Resident DCs were defined as MHC IIint CD11chi cDC1 (CD8+) and cDC2 (Sirp⍰+). For innate immune cells, CD4, CD3, CD8, B220 and CD11c positive cells were excluded in a “dump” channel and cells gated on CD11b+. Neutrophils were defined as Ly6G+ and NK cells as Ly6G- NK1.1+. After gating on Ly6G- NK1.1-, eosinophils were defined as SSC-Hint Ly6C-, Ly6C+ monocytes as SSC-Hlo Ly6C+, Ly6C- and macrophages as SSC-Hlo Ly6C- F4/80+. Counting beads (Accucheck, Thermo Fischer) were added to allow quantification of immune cells. Analysis was performed using LSR Fortessa (BD Biosience).
Instrument	LSR Fortessa BD BioScience
Software	Flow Jo
Cell population abundance	provided in Supplementary Information
Gating strategy	provided in Supplementary Information

☒ Tick this box to confirm that a figure exemplifying the gating strategy is provided in the Supplementary Information.
