# Supplementary material for: In vivo assembly of epitope-coated biopolymer particles that induce anti-tumor responses
Source: NPJ Vaccines. 2024 Jan 23;9:18. doi: 10.1038/s41541-023-00787-8 (PMC10805745; doi:10.1038/s41541-023-00787-8)
Supplement: Supplementary file 2 — Supplemental Material [file 41541_2023_787_MOESM2_ESM.pdf]

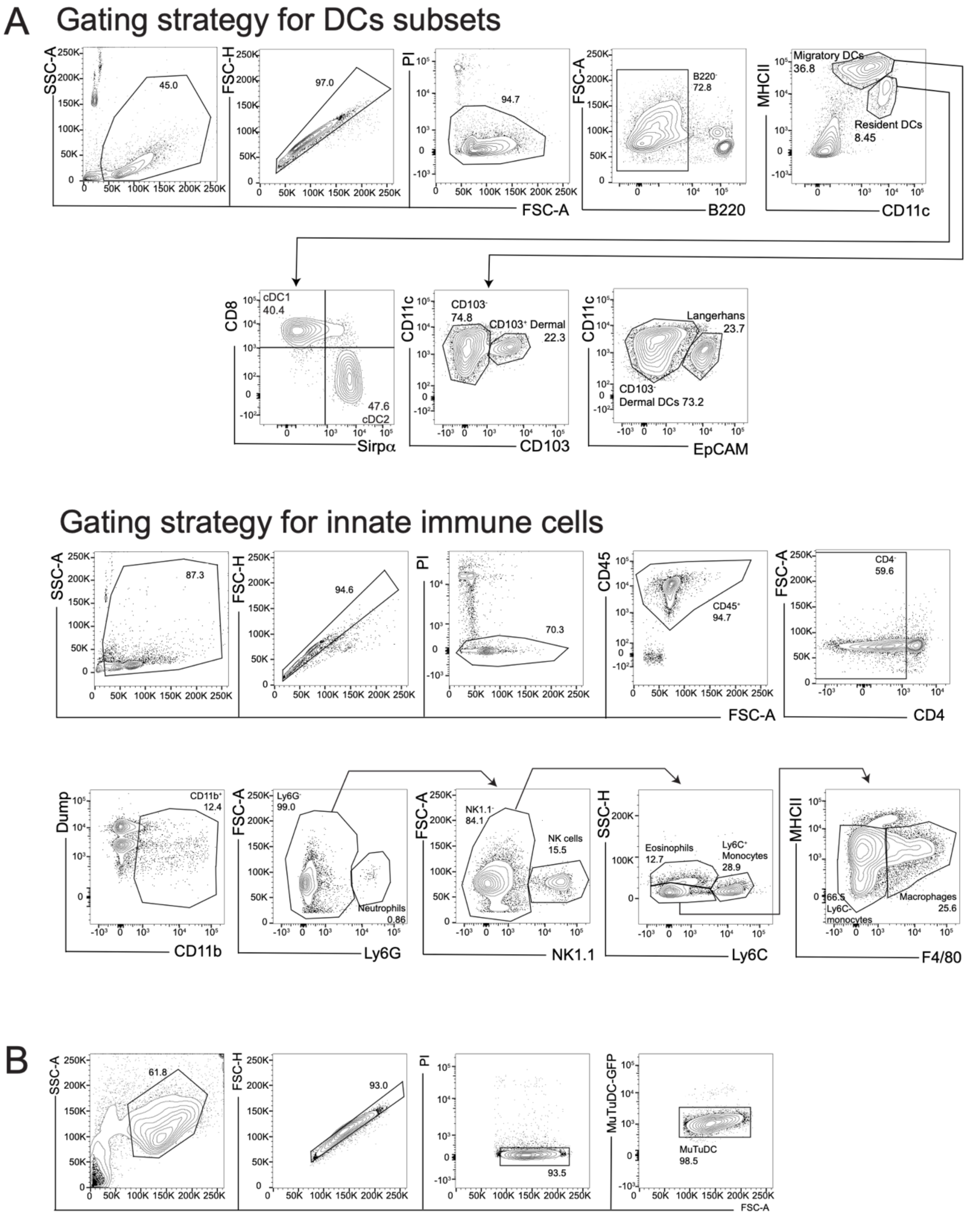


**Supplementary Figure 1. Flow cytometry gating strategies for immune cells. (A)** DCs

subsets and innate immune cells in the ILN**.** C57BL/6 mice were vaccinated subcutaneously with 5 mg of BP-OVA. ILN were harvested 16 hours post-vaccination to obtain single cell suspensions. (**B**) MuTuDCs were incubated with or without 0.5 μM of CpG type B 1688 or 100 μg PHB beads for 24 hours at 37$^{o}$C prior to staining for surface levels of CD86 and MHCII.

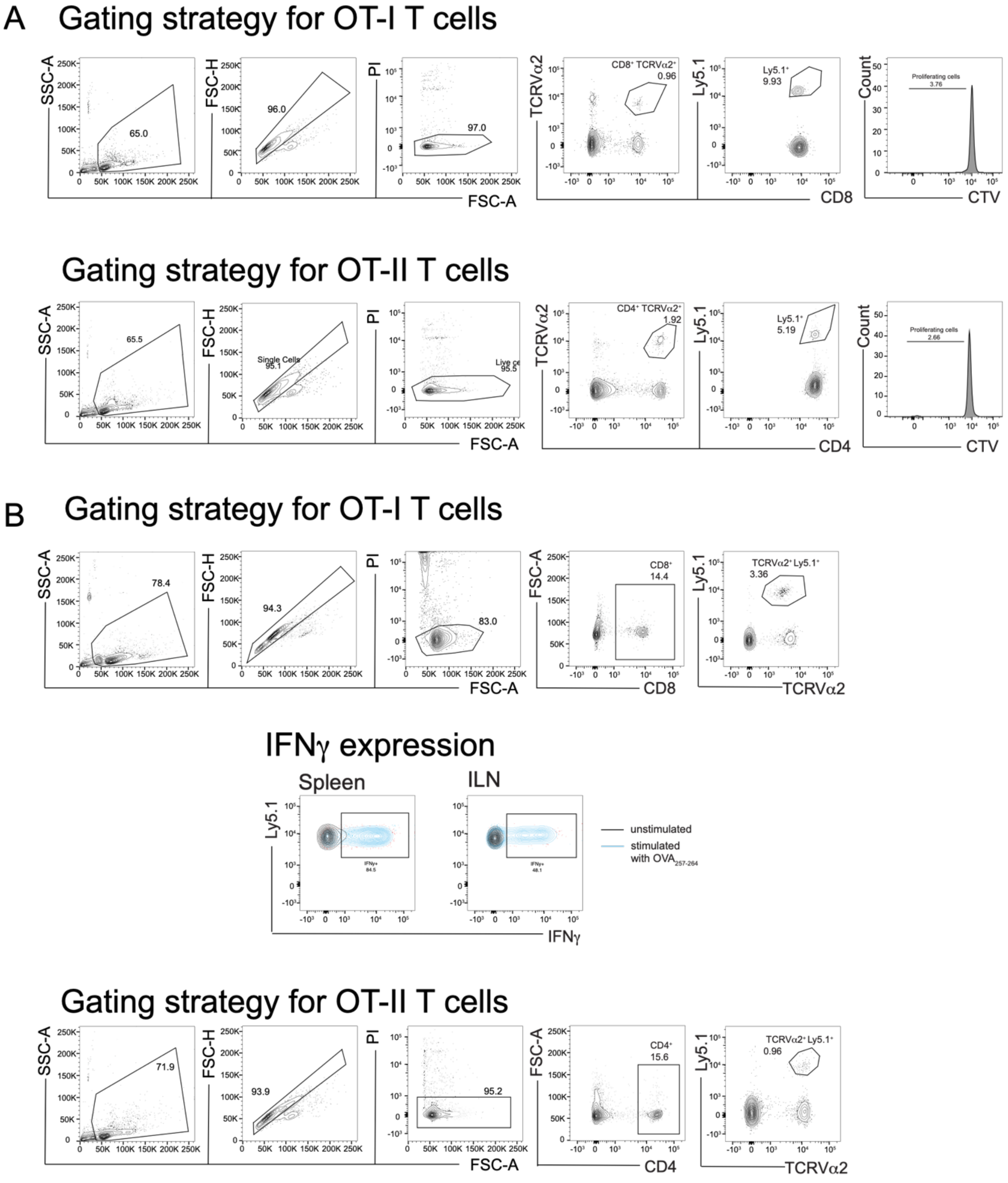


**Supplementary Figure 2**. **Flow cytometry gating strategies for OT-I and OT-II T cells**. **(A)** Gating strategy to identify proliferating OT-I and OT-II T cells for *in vivo* antigen presentation assay analysis. **(B)** Gating strategy for enumeration of percentage, number and PD-1 expression

of OT-I and OT-II T cells after 3, 5, 7, 10, 13, 18 and 21 days post-vaccination. Representative histogram shows IFNγ-producing OT-I T cells in the spleen and ILN.

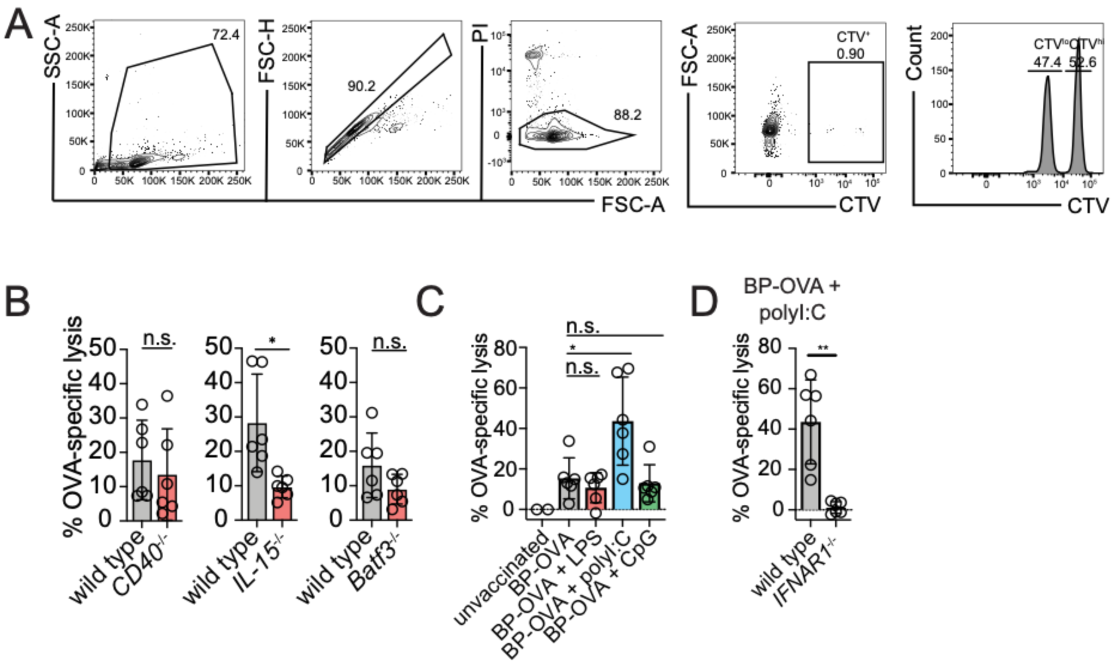


**Supplementary Figure 3. CTL response after subcutaneous BP-OVA vaccination**. (**A**) Gating strategy to obtain CTV$^{+}$ target cells in single cell suspension. (**B**) C57BL/6 (WT), *MHC II*$^{-/-}$. *CD40*$^{-/-}$, *IL-15*$^{-/-}$ and *Batf3*$^{-/-}$ mice were subcutaneously injected with 5 mg of BP-OVA. (**C**) WT mice were injected with BP-OVA combined with 1 µg of LPS, 20 µg polyI:C or 20 nmol of CpG. (**D**) WT mice and *IFNAR1*$^{-/-}$ mice were injected with 5 mg of BP-OVA in the absence of presence of 20µg polyI:C subcutaneously. (**B-D**) Mice would then intravenously be injected with equal number of CTV$^{high}$ (OVA$_{257-264}$$^{+}$) and CTV$^{low}$ (OVA$^{-}$) cell populations six days post-vaccination. Their ILN were harvested 36-42 hours post target cells injection. Histogram represents the % of OVA-specific lysis after vaccination. Two to three independent experiments were performed. Bars

represent ± SD (n=1). *p<0.05, **p<0.01, ***p<0.001, ****p<0.0001, ns=not significant. LPS = polysaccharide, poly I:C = polyinosinic polycytidylic acid.

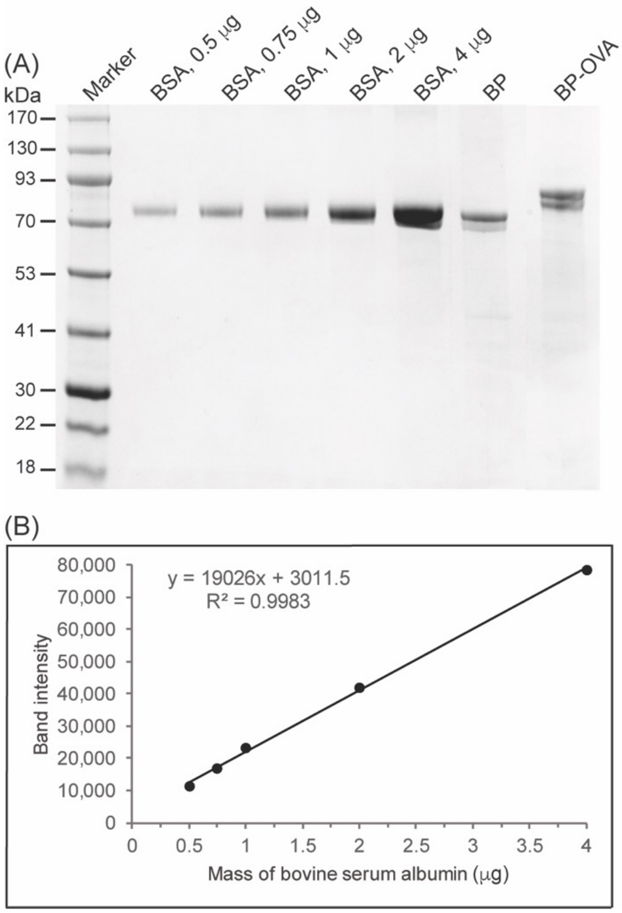


**Supplementary Figure 4. Densitometry analysis for protein quantification using sodium dodecyl sulphate polyacrylamide gel electrophoresis (SDS-PAGE).** (**A**) SDS-PAGE gel image of protein samples at varying dilution factors. (**B**) BSA standard curve used for protein quantifications calculated based on the ImageJ software. Note: The Marker is GangNam-STAIN$^{TM}$ Prestained Protein Ladder (iNtRON Biotechnology, South Korea. Abbreviation: BSA, bovine serum albumin.

**Supplementary Table 1. Bioprocessing yields of PBs from cell cultures and their physicochemical properties, including theoretical molecular weight ($M_w$) of displayed fusion proteins, quantities of fusion proteins, target peptides and PHB.**

| | BPs | BP-OVA |
|---|---|---|
| Mw, fusion protein (kDa) | 65.60 | 74.61 |
| Cell weight* (g) per culture volume (L) | 6.70 | 4.32 |
| Bead weight** (g) per culture volume (L) | 0.35 | 0.22 |
| [1]Fusion protein weight (mg) per bead weight** (g) | 4.58 | 2.77 |
| [1]OVA peptide weight (mg) per bead weight** (g) | - | 0.5650 |
| [2]Poly(3-hydroxybutyrate) weight (mg) per bead weight** (g) | 82.50 | 69.60 |

*Wet cell weight, **Wet bead weight

[1]Measured based on densitometry analyses on SDS-PAGE

[2]Measured using HPLC on acid-digested beads

**Supplementary Table 2. Bacterial strains and plasmids used in this study.**

| **Strains/plasmids** | **Characteristics** | **References** |
|---|---|---|
| *Escherichia coli* strains | | |
| XL1-Blue | *recA1 endA1 gyrA96 thi-1 hsdR17 supE44 relA1 lac* [F′*pro*AB *lacI*$^q$ *lacZ*Δ*M15* Tn*10* (Tet$^R$)] | Stratagene (San Diego, CA) |
| *ClearColi*™ | F$^-$ *ompT hsdS$_B$* ($r_B^-$ $m_B^-$) *gal dcm lon* λ (DE3 [*lacI lac*UV5-T7 gene 1 *ind1 sam7 nin5*]) *msbA148* Δ*gutQ* Δ*kdsD* Δ*lpxL* Δ*lpxM* Δ*pagP* Δ*lpxP* Δ*eptA* | Lucigen (Middleton, WI) |
| Plasmids | | |
| pET-14b | Ap$^r$; T7 promoter | Novagen (Germany) |
| pMCS69 | CM$^r$; T7 promoter, pBBR1MCS derivative containing genes *phaA* and *phaB* from *Cupriavidus necator*, co-linear to *lac* promoter | (1) |
| pET-14b-PhaC | pET-14b containing *phaC* fragment gene | (2) |

| | | |
|---|---|---|
| pUC57-OVA | pUC57 derivative containing *Xba*I/*Not*I fragment gene *ova* | Biomatik (Canada) |
| pET-14b-OVA-PhbC | pET-14b-PhaC derivative containing *BP-OVA* fused to the 5' end of *phaC* | This study |

**Supplementary Table 3. Identification of the fusion proteins based on peptide mass fingerprinting.**

| Fusion proteins displayed on the beads | Peptide fragments were identified by mass spectroscopy and shown within the full-length fusion protein (highlighted in bold) |
|---|---|
| BP-OVA<br><br>(top band) | M **S I I N F E K L GPGPG S I I N F E K L GPGPG S I I N**<br>**F E K L GPGPG I S Q A V H A A H A E I N E A G R GPGPG**<br>**I S Q A V H A A H A E I N E A G R GPGPG I S Q A V H A**<br>**A H A E I N E A G R GPGPG A T G K G A A A S T Q E G K**<br>**S Q P F K V T P G P F D P A T W L E W S R Q W Q G T E**<br>**G N G H A A A S G I P G L D A L A G V K I A P A Q L G**<br>**D I Q Q R Y M K D F S A L W Q A M A E G K A E A T G P**<br>**L H D R R F A G D A W R T N L P Y R F A A A F Y L L N**<br>**A R A L T E L A D A V E A D A K T R** Q R I **R F A I S Q**<br>**W V D A M S P A N F L A T N P E A Q R L L I E S G G E**<br>**S L R A G V R N M M E D L T R G K I S Q T D E S A F E**<br>**V G R N V A V T E G A V V F E N E Y F Q L L Q Y K P L** |

| | |
|---|---|
| | **T D K V H A R P L L M V P P C I N K Y Y I L D L Q P E**<br>**S S L V R H V V E Q G H T V F L V S W R N P D A S M A**<br>**G S T W D D Y I E H A A I R A I E V A R D I S G Q D K**<br>**I N V L G F C V G G T I V S T A L A V L A A R G E H P**<br>**A A S V T L L T T L L D F A D T G I L D V F V D E G H**<br>**V Q L R E A T L G G G A G A P C A L L R G L E L A N T**<br>**F S F L R P N D L V W N Y V V D N Y L K G N T P V P F**<br>**D L L F W N G D A T N L P G P W Y C W Y L R H T Y L Q**<br>**N E L K V P G K L T V C G V P V D L A S I D V P T Y I**<br>**Y G S R E D H I V P W T A A Y A S T A L L A N K L R F**<br>**V L G A S G H I A G V I N P P A K** N K R S H W T N D A<br>L P E S P Q Q W L A G A I E H H G S W W P D W T A W L<br>A G Q A G A K **R A A P A N Y G N A R** Y R **A I E P A P G**<br>**R** Y V K A K A |
| BP-OVA<br><br>(bottom band) | M **S I I N F E K L GPGPG S I I N F E K L GPGPG S I I N**<br>**F E K L GPGPG I S Q A V H A A H A E I N E A G R GPGPG**<br>**I S Q A V H A A H A E I N E A G R GPGPG I S Q A V H A**<br>**A H A E I N E A G R GPGPG A T G K G A A A S T Q E G K**<br>**S Q P F K V T P G P F D P A T W L E W S R Q W Q G T E**<br>**G N G H A A A S G I P G L D A L A G V K I A P A Q L G**<br>**D I Q Q R Y M K D F S A L W Q A M A E G K A E A T G P**<br>**L H D R R F A G D A W R T N L P Y R F A A A F Y L L N**<br>**A R A L T E L A D A V E A D A K T R Q R** I R **F A I S Q**<br>**W V D A M S P A N F L A T N P E A Q R L L I E S G G E**<br>**S L R A G V R N M M E D L T R G K I S Q T D E S A F E**<br>**V G R N V A V T E G A V V F E N E Y F Q L L Q Y K P L** |

| | |
|---|---|
| | **T D K V H A R P L L M V P P C I N K Y Y I L D L Q P E**<br>**S S L V R H V V E Q G H T V F L V S W R N P D A S M A**<br>**G S T W D D Y I E H A A I R A I E V A R D I S G Q D K**<br>**I N V L G F C V G G T I V S T A L A V L A A R G E H P**<br>**A A S V T L L T T L L D F A D T G I L D V F V D E G H**<br>**V Q L R E A T L G G G A G A P C A L L R G L E L A N T**<br>**F S F L R P N D L V W N Y V V D N Y L K G N T P V P F**<br>**D L L F W N G D A T N L P G P W Y** C W Y L R **H T Y L Q**<br>**N E L K V P G K L T V C G V P V D L A S I D V P T Y I**<br>**Y G S R E D H I V P W T A A Y A S T A L L A N K L R F**<br>**V L G A S G H I A G V I N P P A K** N K R S H W T N D A<br>L P E S P Q Q W L A G A I E H H G S W W P D W T A W L<br>A G Q A G A K **R A A P A N Y G N A R** Y R **A I E P A P G**<br>**R Y** V K A K A |

**Supplementary Table 4. Summary of the chromatography system suitability test results using adipic acid as the internal standard and crotonic acid (CA) as the standard for quantification of poly(3-hydroxybutyrate) (PHB) from the beads (BP, BP-OVA).**

| Sample | Adipic acid | | | Crotonic acid | | |
|---|---|---|---|---|---|---|
| | Retention time (min) | Area | Amount (g/L) | Retention time (min) | Area | Amount (g/L) |
| Crotonic acid (CA) standard | | | | | | |
| CA, 0.006 mg/L | 16.143 | 10.687 | 0.2 | 26.36 | 0.039 | 0 |

| | | | | | | |
|---|---|---|---|---|---|---|
| CA, 0.012 mg/L | 16.143 | 10.740 | 0.2 | 26.427 | 0.068 | 0 |
| CA, 0.024 mg/L | 16.143 | 10.739 | 0.2 | 26.380 | 0.149 | 0.0001 |
| CA, 0.049 mg/L | 16.143 | 10.758 | 0.2 | 26.260 | 0.298 | 0.0001 |
| CA, 0.098 mg/L | 16.143 | 10.777 | 0.2 | 26.377 | 0.578 | 0.0001 |
| CA, 0.195 mg/L | 16.143 | 10.811 | 0.2 | 26.370 | 1.150 | 0.0002 |
| CA, 0.391 mg/L | 16.143 | 10.814 | 0.2 | 26.370 | 2.271 | 0.0004 |
| CA, 0.781 mg/L | 16.143 | 10.749 | 0.2 | 26.370 | 4.567 | 0.0008 |
| CA, 1.563 mg/L | 16.143 | 10.683 | 0.2 | 26.373 | 9.228 | 0.0016 |
| CA, 3.125 mg/L | 16.143 | 10.751 | 0.2 | 26.370 | 18.333 | 0.0031 |
| CA, 6.25 mg/L | 16.143 | 10.781 | 0.2 | 26.370 | 37.337 | 0.0063 |
| CA, 12.5 mg/L | 16.143 | 10.814 | 0.2 | 26.370 | 73.24 | 0.0124 |
| CA, 25 mg/L | 16.143 | 10.780 | 0.2 | 26.370 | 146.756 | 0.0248 |
| CA, 50 mg/L | 16.143 | 10.760 | 0.2 | 26.370 | 294.354 | 0.0498 |
| CA, 100 mg/L | 16.143 | 10.662 | 0.2 | 26.377 | 592.282 | 0.1002 |
| PHB Samples | | | | | | |

| | | | | | | |
|---|---|---|---|---|---|---|
| BP, 95 mg* | 16.147 | 10.469 | 0.2 | 26.370 | 442.331 | 0.0748 |
| BP-OVA, 19 mg* | 16.147 | 10.543 | 0.2 | 26.373 | 77.345 | 0.0131 |

*weight after freeze drying used for digestion with $H_2SO_4$.
